# Characterization of Histone Deacetylase Mechanisms in Cancer Development

**DOI:** 10.3389/fonc.2021.700947

**Published:** 2021-07-29

**Authors:** Rihan Hai, Liuer He, Guang Shu, Gang Yin

**Affiliations:** ^1^Department of Pathology, Xiangya Hospital, School of Basic Medical Sciences, Central South University, Changsha, China; ^2^School of Basic Medical Sciences, Central South University, Changsha, China

**Keywords:** epigenetics, protein acetylation, histone deacetylases, cancer, tumorigenesis

## Abstract

Over decades of studies, accumulating evidence has suggested that epigenetic dysregulation is a hallmark of tumours. Post-translational modifications of histones are involved in tumour pathogenesis and development mainly by influencing a broad range of physiological processes. Histone deacetylases (HDACs) and histone acetyltransferases (HATs) are pivotal epigenetic modulators that regulate dynamic processes in the acetylation of histones at lysine residues, thereby influencing transcription of oncogenes and tumour suppressor genes. Moreover, HDACs mediate the deacetylation process of many nonhistone proteins and thus orchestrate a host of pathological processes, such as tumour pathogenesis. In this review, we elucidate the functions of HDACs in cancer.

## Background

Epigenetics refers to molecular processes that regulate gene expression without altering the DNA sequence, including diverse molecular modifications of DNA and chromatin, such as histone acetylation and DNA methylation (1, 2). Epigenetics can turn on/off gene expression and thus plays a crucial role in tumorigenesis and cancer progression (3). Abnormal epigenetic alterations and destroyed epigenetic integrity are common characteristics of tumour cells (4). Among all epigenetic mechanisms, histone modifications have been shown to be important in carcinogenesis.

Histone modifications are catalysed by specific enzyme complexes through ATP-consuming processes, which in turn impact gene transcription, duplication, repair and recombination (4). Acetylation and methylation are involved in the regulation of amino-terminal (N-terminal) tail domains of core histones and affect the post-translational crosstalk between DNA and histones by recruiting proteins and transcription factors (5).

**Figure 1:**
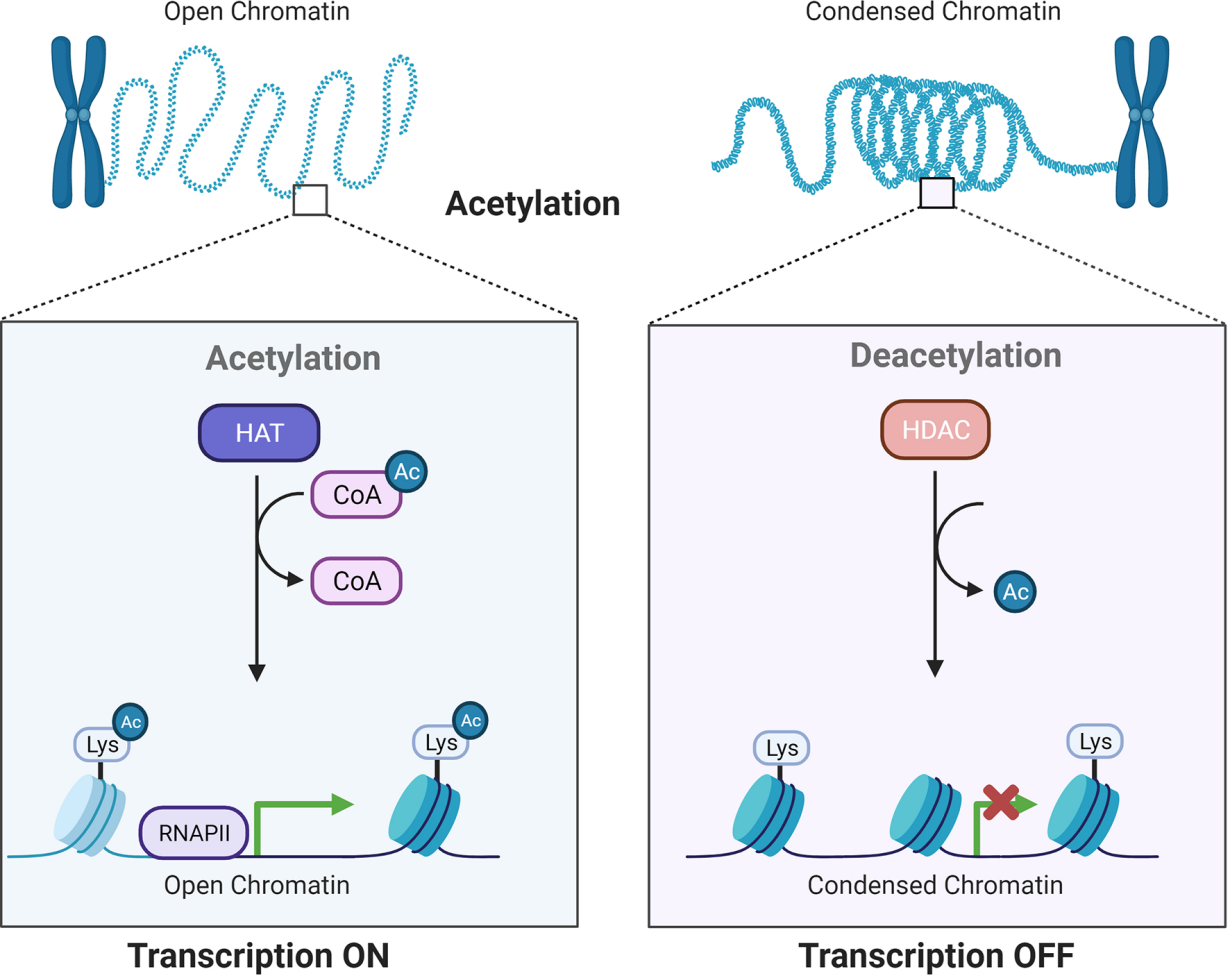
Acetylation by HATs and deacetylation by HDACs influence gene transcriptional activity. HATs and HDACs add or remove acetyl groups at the N−terminus lysine, which leads to an open or condensed state of the chromatin.

Histone acetylation involves reversible alterations of the N-terminal lysine on histones and is subsequently linked to different cellular processes and disease development (6, 7). Histone acetylation is accomplished by two enzymes: histone acetyltransferases (HATs) and histone deacetylases (HDACs or KDACs), which add or remove acetyl groups (8) (**Figure 1**). The existence of acetylated lysine in the histone tail causes the chromatin to be more open, allowing initiation of gene transcription. In contrast, deacetylation enhances the ionic interactions between histones, which have a positive charge, and DNA, which has a negative charge. Deacetylation is related to compacted chromatin, which is not conducive to gene transcription (9). These modifications show strong relationships to gene expression in normal and cancerous cells (10).

HDACs act as gene silencing complexes, and studies have suggested that HDACs suppress gene expression through transcription factors such as E2F1. Furthermore, evidence indicates that HDACs can eliminate acetylation of nonhistone proteins (11). HDACs inhibit the process by which T cells recognize and destroy tumour cells (12).

## HDAC Classifications

**Figure 2:**
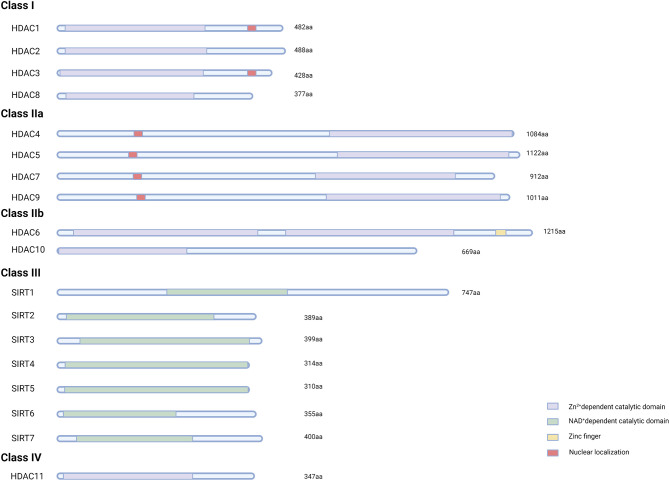
The domain structure of human HDACs. The basic domain structure of eighteen HDAC members. Class I, II, and IV HDACs contain Zn^2+-^dependent domains, while SIRTs contain NAD^+-^dependent domains. Nuclear localization sequences and zinc finger motifs in different HDACs are also shown.

Human HDACs can be classified into four classes with 18 members (**Figure 2**). Class I and II HDACs were found to show high similarities in catalytic sites. Except for HDAC8, HDAC isoforms appear to combine with more proteins or more HDAC isoforms in multiprotein complexes (13, 14). Many types of HDACs are derived from nucleotide polymorphisms and optional splicing. Various isoforms of HDAC9 have been identified (15).

### Class I HDACs

These enzymes have been identified in the nucleus. The expression of class I HDACs is elevated in cancer cells. HDAC1 mainly shows oncogenic activity, but a dual role of HDAC1 was found in different stages of acute promyelocytic leukaemia (APL) (16). HDAC1 functions as a tumour suppressor by restraining the activity of PML-RAR in the preleukaemic stage of APL. Moreover, it exerts tumour-promoting activity in established tumour cells (16). This finding reveals the importance of identifying the role of HDACs at different stages in different cancer cell types for optimal treatment, such as personalized design of inhibitors.

HDAC1 and HDAC2 are involved in the deacetylation of p53, which results in enhanced regulation of target genes by p53 (17, 18). The interaction between HDAC3 and cancer-associated genes influences the angiogenic and carcinogenic potential of tumours and the efficacy of antitumour drug treatment (19). Furthermore, HDAC3 can catalyse the deacetylation of the Notch1 intracellular domain (NICD1), thereby promoting NICD1 protein stability, which is involved in the progression of T cell acute lymphoblastic leukaemia (ALL) and chronic lymphocytic leukaemia (CLL) (20). In addition, HDAC3 inhibits NF-κB lysine acetylation, thereby exerting a proinflammatory effect (21).

When the expression of HDAC1 is decreased, the levels of HDAC2 and HDAC3 are increased. However, highly expressed HDAC2 and HDAC3 are unable to compensate for the lack of HDAC1 (22).

HDAC8 is overexpressed in diverse cancer tissues, including colon, breast, lung, pancreatic, and liver cancers and childhood neuroblastoma (23). The distal region of HDAC8 allosterically regulates the activity of this enzyme (24). In addition, oxidoreduction reactions can modulate the activity of HDAC8 (25).

#### The HDAC1/2 Complex

Homologous proteins of class I HDAC can form stable multiprotein complexes with other proteins. Over the years, several HDAC1/2 corepressor complexes, such as switch-independent protein 3A (SIN3A), mitotic deacetylase (MiDAC), nucleosome remodelling and deacetylase (NuRD), mesoderm induction early response (MIER), corepressor of REST (CoREST), and arginine-glutamic acid dipeptide repeats (RERE), have been identified (26, 27).

The structures of SIN3A and NuRD demonstrated the diversity of protein complex components, which are related to their function and context-specific cellular activity (28).

##### The SIN3 Complex

The SIN3 complex is composed of SIN3A/B, HDAC1/2, suppressor of defective silencing 3 (SDS3), and SIN3-associated protein p30 (SAP30) (27). Except for SIN3A/B, all corepressor proteins possess an ELM2–SANT domain. The SIN3A complex has more enzymatic activities based on its functional molecules (29, 30). The SIN3 complexes can also form a dimer (31). The acetylation of STAT3 and its interaction with SIN3A inhibit the expression of tumour suppressor genes (32). Nevertheless, the interaction pattern with the above modulators is still unclear.

Recently, PHF23, an H3K4me3 reader, was shown to directly bind the SIN3-HDAC complex and repress its deacetylation activity. Thus, the PHF23-SIN3-HDAC (PSH) complex consequently enhances the activation of downstream tumour suppressor genes (33). Aberrant PSH levels have a stimulatory role in chromosome 17p-deleted tumours.

##### The NuRD Complex

The NuRD complex consists of chromodomain helicase DNA-binding proteins (CHD3/CHD4/CHD5), HDAC1/HDAC2, metastasis associated proteins (MTA1/MTA2/MTA3), methyl domain binding proteins (MBD2/MBD3), retinoblastoma binding proteins (RBBP4/RBBP7), and GATA zinc finger domain proteins (GATAD2B/GATAD2A) (34, 35), and all NuRD components showed high expression in tumour cells (36).

The NuRD complex is formed by six main protein subunits, which have some functional differences (37). The NuRD complex modulates the process by which different cells read DNA, which can trigger pluripotency in stem cells and is involved in the transformation of adult cells to induced pluripotent stem cells (37, 38). The NuRD complex plays important roles in diverse malignant phenotypes in hepatocellular carcinoma (HCC) cells (36).

### Class II HDACs

Class II HDACs have different effects in different tissues (39). HDAC4, HDAC5 and HDAC7 alter cell differentiation on the basis of certain signals and thus lead to changes in the gene expression status. HDAC4, HDAC5, HDAC7, and HDAC9 (class IIa members) are encoded by various genes (40). The catalytic domains of HDAC6 and HDAC10 (class IIb members) show similarity with those of HDAC9. Furthermore, HDAC9 was reported to have splice variants (26). In addition, phosphorylation of class IIa enzymes is associated with their localization and activities in the nucleus (41). Compared to that of other HDACs, the histone deacetylating activity of class IIa HDACs is low. However, class IIa HDACs exert enzymatic activity when forming protein complexes with SMRT/N-CoR (42). A recent study reported that class IIa HDACs are directly involved in lung vascular barrier disruption (43). Class IIa HDACs may also regulate the endothelial cell barrier through deacetylation, since HDAC7 is closely related to cytoskeletal processes (43).

Class IIa HDACs repress transcriptional processes in diverse tissues. In addition to acting as transcription repressors, class II HDACs are associated with autophagic progression and cytoskeleton microtubules (44). For example, HDAC6 can act as a α-tubulin deacetylase and participate in multiple cytoplasmic pathways related to microtubules (45, 46), revealing that it may be an important therapeutic for treating Alzheimer’s disease and cancer (47). HDAC4 and HDAC9 contain different common genomic binding sites. However, HDAC4 binds additional sites that may escape modulation by HDAC9 (48). HDAC9 inhibits cardiomyocyte hypertrophy and skeletal muscle differentiation. HDAC4 suppresses the activity of Runx2, thereby repressing chondrocyte hypertrophy and endochondral bone formation (49). The expression of HDAC5 and lysine-specific demethylase 1 (LSD1) was elevated in breast cancer, and HDAC5 enhanced the LSD1 protein stability and reduced the nuclear level of H3K4me1/me2 in breast cancer cells (50).

### Class III HDACs

To date, seven Sirtuins have been identified. Sirtuins contain catalytic domains, which function in a nicotinamide adenine dinucleotide (NAD+)-dependent manner in transcriptional processes. Sirtuins have also been reported to have lysine defatty-acylase activity (51).

SIRT1 was shown to have a dual role in tumour cell growth (52, 53). This molecule acts as a positive regulator of proteins involved in tumour suppressor pathways or DNA damage repair (54). SIRT1 also has a negative effect on tumorigenesis since it can decrease oncogene transcriptional activity through interaction and deacetylation of c-MYC (52, 55). SIRT2 was reported to exert tumour repressing effects. The deletion of SIRT2 disrupted the checkpoints of the cell cycle and led to enhanced tumorigenesis (56, 57). In addition to mitosis, SIRT2 can regulate genome integrity (58). SIRT3 was reported to modulate transcription factors in breast cancers (59). In lung cancer cells, SIRT4 suppressed cell proliferation, invasion and migration (60). In liver cancer, SIRT5 enhanced tumour cell proliferation (61). In retinoblastoma, SIRT6 was shown to be a cancer suppressor protein and function by restraining the metabolism of cells (62). SIRT6 can stabilize the normal genome of neighbouring cells (63). Moreover, SIRT7 was observed to target H3 histones with high specificity and recruit RNA polymerase I (64, 65).

### Class IV HDACs

Located in the N-terminal tail, the catalytic domain of HDAC11 is very similar to that of HDAC3 and HDAC8 (66). Similar to Sirtuins, HDAC11 also possesses acylase activity. Determination of defatty-acylase activity is considered an alternative method of identifying HDACs, and zinc-dependent HDACs showed notable differences in activity (67, 68). HDAC11 participates in oligodendrocyte progression and promotes oligodendrocyte differentiation (69). This molecule is also involved in immune responses by decreasing IL-10 levels (68). A recent study found that HDAC11 can promote the malignant phenotypes of JAK2-driven myeloproliferative neoplasms (70).

## Role of HDACs in Tumours

HDACs are involved in various stages of cancer (71). Highly expressed HDACs are usually associated with terminal illness and inferior outcomes of patients. For instance, upregulation of HDAC1, HDAC2, and HDAC3 expression correlated with worse survival in patients with gastric and ovarian tumours (72), and elevated levels of HDAC8 expression in neuroblastoma were related to advanced disease and negative outcomes (73, 74).

Notably, the HDAC expression level may not be a prerequisite of their functions in cancer, since abnormal activities of HDACs in cancer progression are common (75). Furthermore, some HDAC families serve as subunits of large protein complexes and can promote carcinogenesis (76, 77).

Research has shown that histone deacetylase activity is not generally necessary for gene expression. The acetyltransferase activity of these molecules cannot recruit p300/CBP and transcription factors but can facilitate the recruitment of TFIID and RNAPII at virtually all enhancers and enhancer-regulated genes (78). Histone acetylation promotes transcription of paused genes through release of Pol II into elongation (79). Research has shown that global histone acetylation depends on ongoing transcription. For instance, Wang and his colleagues have found that in K562 cells, transcription inhibition leads to rapid loss of H3K27ac from enhancers and promoters. Hos2 histone deacetylase primarily interacts with genes with high genome-wide activity and catalyses deacetylation of lysines in the H3 and H4 histone tails (80). Therefore, whether HDACs have context-specific rather than general functions in modulation of gene expression requires further exploration.

HDAC inhibitors (HDACis) were shown to exert antitumour effects through various mechanisms in several cancer cell lines (81). Different HDACis show distinctive mechanisms in influencing cell growth, apoptosis, migration, and angiogenesis (82, 83). HDACis can be classified into four main types according to their structures: short-chain fatty acids, hydroxamic acids, cyclic peptides, and benzamides (84). In recent years, four HDACis have been officially approved by the FDA for clinical therapy of T-cell lymphoma (TCL) and multiple myeloma: vorinostat, romidepsin, belinostat and panobinostat (11, 85). For example, panobinostat causes G2/M cell cycle arrest and apoptosis due to suppression of HDAC3 and HDAC6 and thus leads to degradation of Aurora A and B kinases (86). Panobinostat also enhanced the expression of CDH1 and restrained epithelial-mesenchymal transition (EMT) in triple-negative breast cancer (87). Entinostat leads to low Bcl-XL expression and promotes the apoptotic process of tumours. Entinostat was also shown to cause G1 cell cycle arrest by upregulating p21 expression (88).

Generally, the anticancer activity of HDACis indicates the tumour-promoting effects of HDACs. However, genetic inactivation of HDACs might play a tumorigenic role. HDAC1 somatic mutations and HDAC4 homozygous deletions were observed in 8.3% of dedifferentiated liposarcomas (89) and 4% of melanomas (90). In addition, HDAC2 has been shown to exert an anticancer effect *in vitro* and *in vivo*. Class II HDACs may also serve as cancer suppressors in specific cellular settings. HDAC6 showed low expression in liver cancer cells and liver transplantation patients. Furthermore, the status of HDAC6 is related to poor prognosis of disease. For class III HDACs, a SIRT6 mutation, which is a loss-of-function mutation, was observed in tumour cells and promoted tumour formation (91).

## Possible Mechanisms of HDACS in Cancer Development

**Figure 3:**
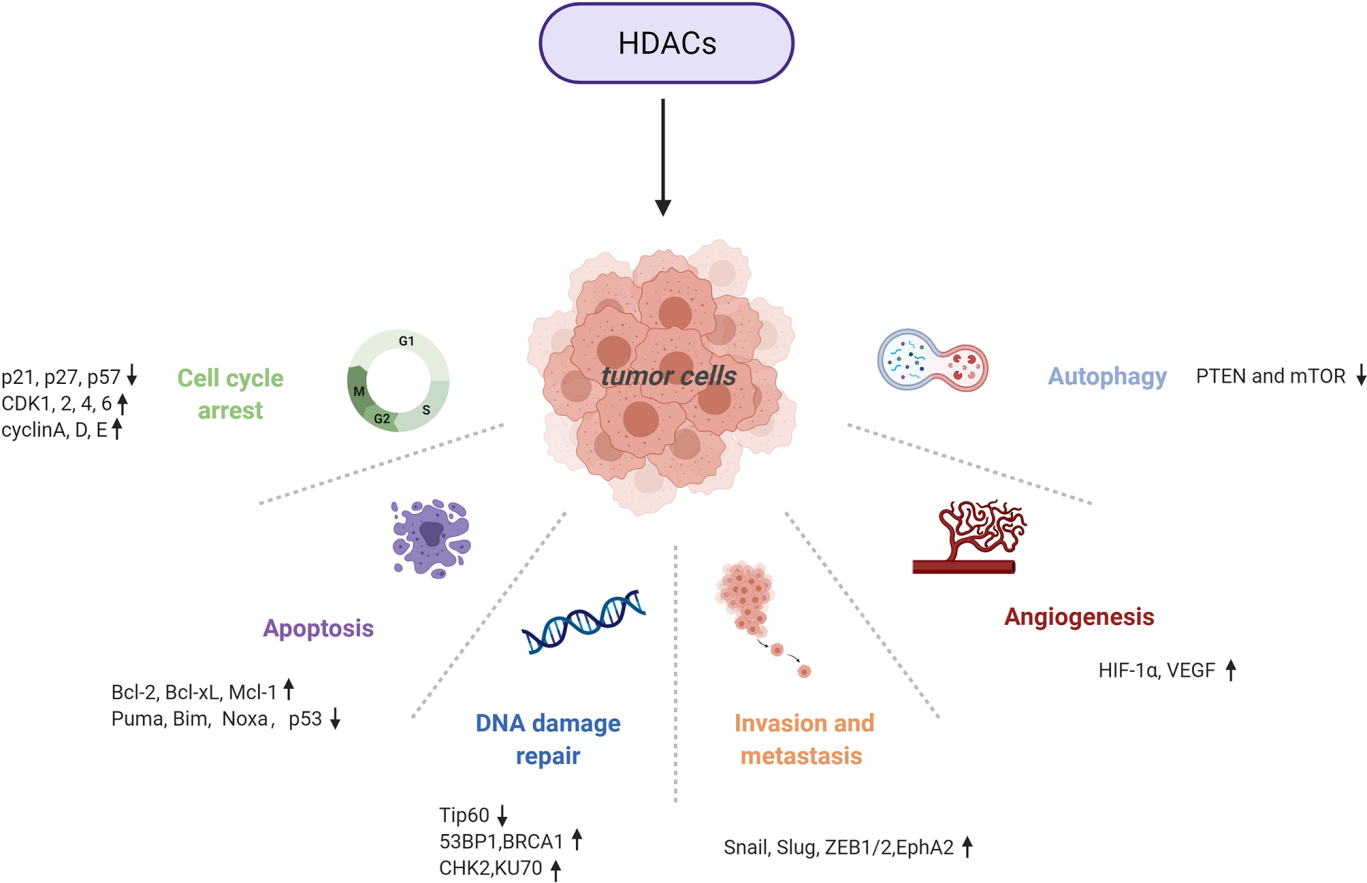
Multiple tumorigenic pathways activated by HDACs. An overview of HDAC-involved tumorigenic processes, including the cell cycle, apoptosis, DNA damage repair, metastasis, angiogenesis, and autophagy.

**Figure 4:**
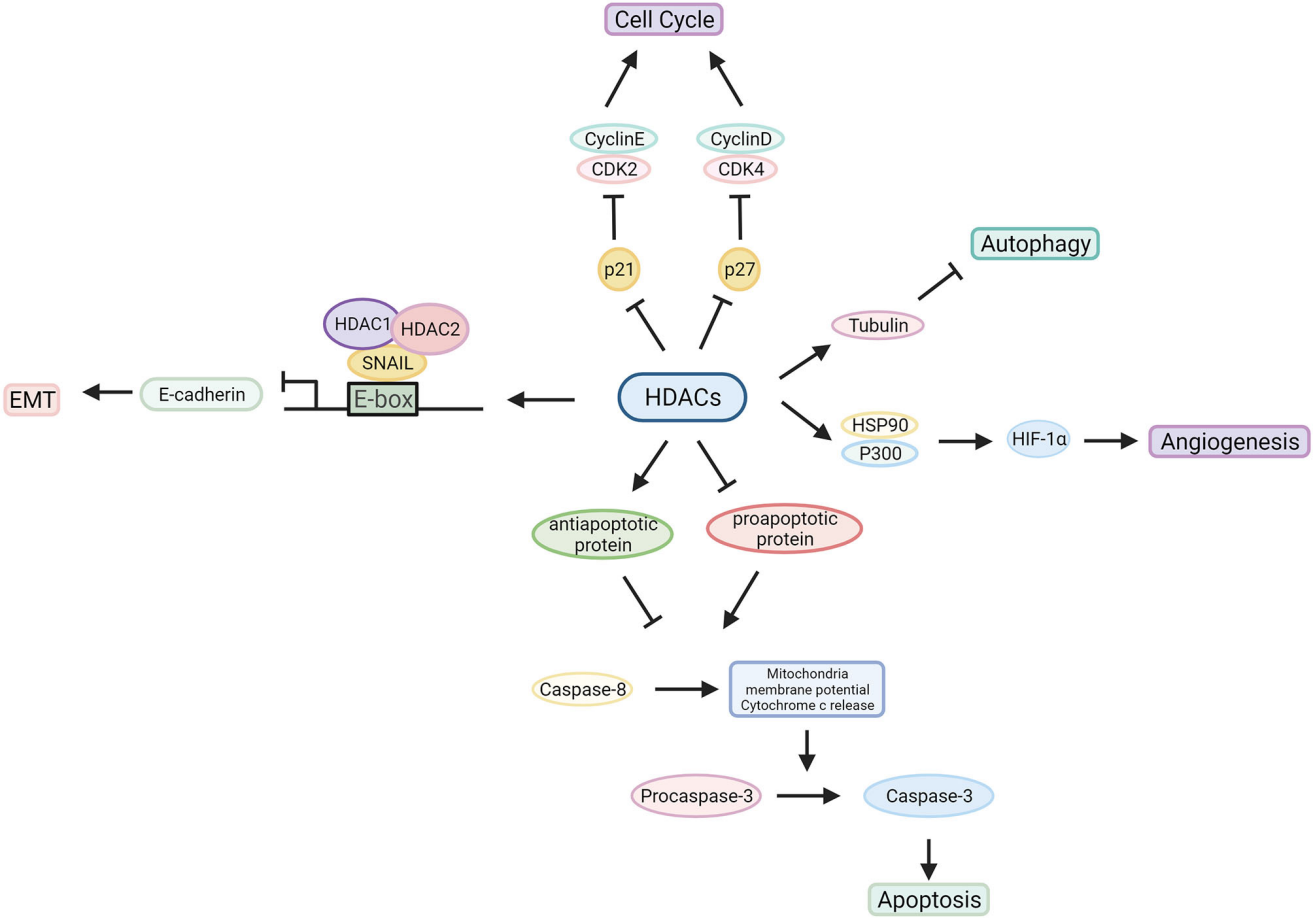
Characterized mechanism of HDACs in cancer development. HDACs influence the tumorigenic process by regulating oncogene and tumour suppressor gene expression or interacting with related proteins.

HDACs are involved in tumour pathogenesis and progression by deacetylating histone and nonhistone proteins that participate in the modulation of multiple tumorigenic pathways (92) (**Figures 3**, **4** and **Table 1**).

**Table 1:** Summary of histone deacetylases.

Super Family	Class	Subclass	Type	Localization	Amino Acid	Cancer Type	Biological relevance	Molecular mechanism
Arginase/deacetylase superfamily	Class I		HDAC1	Nucleus	482	Elevated in lung, liver, breast, colorectal, prostate, ovarian, bladder, gastric, renal, and haematological cancers, HL, and APL	Promotes cell cycle progression and cell proliferation; inhibits apoptosis (11)	KD of HDAC1 upregulates miR-449 and downregulates c-MET expression, which inhibits tumour growth; KD of HDAC1 promotes p21 and p27 expression, inhibits cyclin D1 and CDK2 expression, and reduces cell proliferation by downregulating cyclin A expression (93–95)
Arginase/deacetylase superfamily	Class I		HDAC2	Nucleus	488	Elevated in gastric, prostate, colorectal, pancreatic, breast, renal, medulloblastoma, and bladder cancers, ALL, CTCL and HL	Promotes cell cycle progression and cell proliferation; inhibits apoptosis (11, 96)	KD of HDAC2 upregulates miR-449 and downregulates c-MET expression, which inhibits tumour growth; KD of HDAC2 activates p53 and Bax but suppress Bcl-2 to induce apoptosis; KD induces expression of p21 and suppresses cyclin E2, cyclin D1, and CDK2 expression to induce cell-cycle arrest; KD HDAC2 increases expression of NOXA, which sensitizes tumour cells towards etoposide-induced apoptosis (94, 96, 97)
Arginase/deacetylase superfamily	Class I		HDAC3	Nucleus and cytoplasm	428	Elevated in gastric, breast, colorectal, lung, ovarian, renal, prostate, and bladder cancers, melanoma, ALL and HL	Promotes cell proliferation; inhibits differentiation and apoptosis (11)	KD of HDAC3 upregulates miR-449 and downregulates c-MET expression, which inhibits tumour growth; KD of HDAC3 upregulates E-cadherin expression and reduces cell migration; HDAC3 interacts with ERα and form the HDAC3-ERα complex, which suppresses selective apoptosis mediated by TNF-α (93, 94, 98)
Arginase/deacetylase superfamily	Class I		HDAC8	Nucleus, mitochondria and cytoplasm	377	Elevated in neuroblastoma, melanoma, ALL	Promotes proliferation; inhibits differentiation and apoptosis (11)	HDAC8 inhibition induces p21^WAF1/CIP1^ and NTRK1/TrkA gene expression, which regulates cell cycle arrest and differentiation; HDAC8 regulates CREB, thereby promoting retinoic acid-mediated differentiation (73, 74)
Arginase/deacetylase superfamily	Class II	Class IIa	HDAC4	Nucleus and cytoplasm	1084	Elevated in ALL	Promotes angiogenesis; inhibits differentiation (11)	HDAC4 reduces the level of cleaved caspases 3 and 9 (99)
Arginase/deacetylase superfamily	Class II	Class IIa	HDAC5	Nucleus and cytoplasm	1122	Elevated in medulloblastoma, ALL; decreased in lung cancer	Inhibits migration and differentiation (11)	HDAC5 interacts with LSD1; promotes cell proliferation by upregulating Six expression; KD of HDAC5 promotes apoptosis by upregulating cyto C, caspase 3, p53, and bax expression and induces G1 phase cell-cycle arrest by increasing p21 expression and decreasing cyclin D1 and CDK2/4/6 expression (50, 100, 101)
Arginase/deacetylase superfamily	Class II	Class IIa	HDAC7	Nucleus and cytoplasm	912	Elevated in ALL, CLL; decreased in lung cancer	Promotes angiogenesis (11)	Carboxyl terminus of HDAC7 has sufficient activity for transcriptional repression; HDAC7 can repress transcription through deacetylation-dependent and -independent mechanisms (102)
Arginase/deacetylase superfamily	Class II	Class IIa	HDAC9	Nucleus and cytoplasm	1011	Elevated in ALL, CLL; decreased in medulloblastoma	Promotes angiogenesis (11)	Splice variants with catalytic domain on its C-terminus (103)
Arginase/deacetylase superfamily	Class II	Class IIb	HDAC6	Cytoplasm	1215	Elevated in CTCL, CLL, breast cancer; decreased in lung cancer	Promotes and inhibits angiogenesis, promotes migration (11, 104, 105)	promotes the recruitment of HSP90 and p300 to enhance HIF-1’s transcriptional activity; KD of HDAC6 upregulates the HIF-1α and VEGFA expression, thereby facilitating angiogenesis; HDAC6 interacts with CLIP-170 and promotes tumour cell migration (104, 105)
Arginase/deacetylase superfamily	Class II	Class IIb	HDAC10	Cytoplasm	669	Elevated in CLL	Promotes angiogenesis; inhibits metastasis (11, 106)	HDAC10 regulates autophagy and cytotoxic drug resistance by interacting with HSP70; HDAC10 silences MMP2 and MMP9 expression, thereby inhibiting tumour cell migration and invasion; regulates cyclinA2, thereby affecting the cell cycle (106–108)
Arginase/deacetylase superfamily	Class IV		HDAC11	Nucleus	347	Elevated in breast, renal, and liver cancers	Promotes proliferation, metastasis, and cell cycle; inhibits apoptosis (70, 109–111)	interacts with HDAC1/2; defatty-acylate substrate activity (67, 68, 103)
Deoxyhypusine synthase like NAD/FAD-binding domain superfamily	Class III	I	SIRT1	Nucleus	747	Elevated in CLL; decreased in breast, bladder, prostate, brain, and ovarian cancers, colon carcinoma, OSCC, glioblastoma	Promotes metastasis, autophagy, chromatin stability; disturbs cell cycle and angiogenesis; influences DNA repair, chemoresistance, metabolism, stress response (113, 114, 117–119)	Suppresses p53 activity and maintain the cell cycle and proliferation; deacetylates FOXO1, 3, and 4, resulting in transcriptional repression of proapoptotic genes and upregulation of the expression of stress-related genes; regulates autophagy through the SIRT1-FOXO1-Rab7 axis (112–116)
Deoxyhypusine synthase like NAD/FAD-binding domain superfamily	Class III	I	SIRT2	Cytoplasm	389	Elevated in brain cancer; decreased in breast, liver, and prostate cancers, glioblastoma	Promotes metastasis, autophagy; regulates chromatin condensation, DNA repair, cell cycle, metabolism, differentiation (124, 125)	Binds to and activates transcription factor p300, which form the preinitiation complex with, FOXO1 and FOXO3; increases FOXO1’s interaction with PPAR and thus represses PPAR target genes; regulates the activity of cytosolic proteins LDH-A (120–123)
Deoxyhypusine synthase like NAD/FAD-binding domain superfamily	Class III	I	SIRT3	Mitochondria	399	Decreased in breast, ovarian, lung, and prostate cancers, medulloblastoma	Promotes autophagy, apoptosis; regulates DNA repair, metabolism; maintains mitochondrial protein synthesis (130–133)	Activates PI3K/Akt pathway; controls the ATP synthesis through AMPK pathway; suppresses EMT and migration through the Sirt3-Foxo3a pathway, interacts with miR-19b, LKB1 (126–129)
Deoxyhypusine synthase like NAD/FAD-binding domain superfamily	Class III	II	SIRT4	Mitochondria	314	Decreased in gastric, bladder, breast, and lung cancers, leukaemia	Promotes genomic stability; represses tumorigenesis; regulates amino acid catabolism (13, 135)	Downregulates the tumour suppressor PTEN and mTOR thus increasing autophagy (134)
Deoxyhypusine synthase like NAD/FAD-binding domain superfamily	Class III	III	SIRT5	Mitochondria	310	Elevated in lung cancer	Promotes cell proliferation and invasion; regulates urea cycle, metabolism (61, 136, 137, 141)	Inhibits GLS and regulates glutamine metabolism thereby affecting the TCA cycle; BAG3 inhibits the formation of the GLS-SIRT5 complex and prevents proteasomal degradation of GLS, thereby promoting autophagy; activates SOD1 and promote tumour growth (136–140)
Deoxyhypusine synthase like NAD/FAD-binding domain superfamily	Class III	IV	SIRT6	Nucleus	355	Elevated in CLL; decreased in pancreatic, colon, and breast cancers, glioblastoma multiforme	Promotes progression, tumour establishment, chromatin and DNA repair; regulates telomeric chromatin (62, 143, 144)	Activates PARP1 to repair DBS damage under oxidative stress; inhibits the transcription of factors NF-κB, HIF-1α and MYC (62, 140, 142)
Deoxyhypusine synthase like NAD/FAD-binding domain superfamily	Class III	IV	SIRT7	Nucleus	400	Elevated in colorectal, breast, and thyroid cancers	Promotes autophagy; inhibits proliferation and migration; regulates rDNA transcription (146)	Deacetylates PAF53, which recruits RNA polymerase I to rDNA promoter (145)

### Cell Cycle

HDACs facilitate the stage-specific development of cancers; for example, HDAC1 reduces cell cycle suppressors by interacting with Rb and influencing E2F1 activity (9). Therefore, HDACis can impede the transition from G1 to S phase by recurring Rb activity *via* dephosphorylation and inhibition of E2F1 activities (147). HDAC inhibition also exerts anticancer effects by blocking the cell cycle, which is achieved by inducing high expression of cyclin-dependent kinase (CDK) inhibitors or low expression of cyclins and CDKs (148). HDAC1 and HDAC2 can bind to genes such as p21^WAF1/CIP1^ and p27^KIP1^, resulting in suppression of their expression (149). Silencing p21 and p27 (CDK inhibitors) led to facilitated cell proliferation. Thus, inhibition of HDAC1 and HDAC2 promotes cell cycle arrest at G1 phase (148).

Along with HDAC1’s effect on the G1/S transition, it is also involved in the G2/M transition. Knocking down HDAC1 in tumour cells partially contributed to G2/M phase arrest (150). Furthermore, in adult neural stem/progenitor cells, HDAC3 was reported to modulate the G2/M transition through alteration of CDK1 expression (151). In addition, HDAC3 contributes to cell progression and proliferation by modulating the signal transducer and activator of transcription3 signalling pathway in liver cancers (152). Moreover, HDAC10 participates in G2/M transition by regulating the transcription of cyclin A2 protein depending on let-7 and HMGA2 (153). HDAC10 plays a crucial role in unchecked cell progression, since deletion of HDAC10 induces blockade at mitotic entry and thus restrains cell proliferation in human lung cancers (154). High expression of Sp1 induced by HDAC1/2/6 facilitates cell division of malignant cells by enhancing BMI1 and hTERT and, more importantly, G2/M progression (155). Furthermore, an HDAC6 inhibitor can lead to G2/M arrest in temozolomide-resistant cells (155).

Members of class III and class IV HDACs also play roles in cell cycle regulation. SIRT1 can disturb the cell cycle by acting on p53 and blocking all p53-dependent pathways (156). HDAC11 was shown to have a negative effect on E2F7 and E2F8, cell cycle suppressors, thus contributing to cancer cell survival within lymph nodes (109).

HDACis can block the cell cycle at G1/S and G2/M phases, which is similar to the gene knockdown results, confirming multiple effects of HDACs during the cell cycle. These results support the promising role of HDACs as targets for treating aberrant tumour cell growth and proliferation.

### Apoptosis

HDACs participate in the extrinsic and intrinsic pathways of apoptosis. For the extrinsic pathway, HDACs can block TRAIL- or TGF-β-mediated pathways (157). For the intrinsic pathway, the HDAC family alters pro- and antiapoptotic proteins. HDACs can inhibit proapoptotic Bcl-2 proteins, such as NOXA and BAX (158, 159), *via* direct acetylation or by nonhistone protein KU70 modification (160). Moreover, HDACs can promote apoptosis in glioblastoma and other cancer types (161).

HDACi treatment can promote cell apoptosis by the extrinsic/intrinsic pathway and by improving the sensitivity of tumour cells (162). In addition, some HDACis have been assessed in preclinical cancer models. Vorinostat and panobinostat, which are nonselective HDACis, inhibited FLIP expression in a c-MYC-mediated manner (163). By suppressing HDAC1 and HDAC2, HDACis impeded the growth of prostate cancer (164). HDACis successfully reduced the level of FLIP and enhanced caspase-8 activity in non-small cell lung cancer (NSCLC). Under HDACis, cells are sensitive to activators such as TRAIL, thereby promoting apoptosis (165). Furthermore, HDAC2 depletion could help sensitize pancreatic cancer cells to TRAIL-induced apoptosis by upregulating the expression of TRAIL receptor DR5 (TRAIL-R2) (166). Regarding the intrinsic pathway, HDAC inhibition could promote apoptosis by downregulating the expression of Bcl-2 proteins, such as Bcl-2, Bcl-xL and Mcl-1 (161), while upregulating the expression of proapoptotic proteins (167), which include Puma, Bim, and Noxa (168).

Professor Wang found that HDAC1, HDAC2 and HDAC3 are related to microcystin-leucine arginine (MC-LR)-induced apoptosis in SD rat testicular cells. Specifically, HDACs were activated by MC-LR and subsequently reduced the acetylation state of normal testicular cells, resulting in cell cycle abnormalities and consequently cell apoptosis. These researchers also reported that TSA could re-establish MC-LR-induced apoptosis and cell cycle arrest (169).

Overexpression of HDAC1, HDAC2 and HDAC8 has been demonstrated to downregulate the expression of p53, a tumour suppressor gene that participates in cell apoptosis, thus resulting in the inhibition of apoptosis (170–172). Lnc-Ip53, a lncRNA that can be transactivated by p53, was reported to interact with HDAC1 and p300, thereby restraining p53 acetylation, reducing p53 activity and subsequently inhibiting apoptosis (173). HDACis, like TSA, stabilized the acetylation caused by p53 and increased PUMA expression by promoting the binding of p53 to the PUMA promoter, thus abrogating resistance to DNA damage-induced cell death in renal cancer (155, 174). Along with HDAC1, HDAC2 and HDAC8 (50), HDAC3 can interact with oestrogen receptor-α (ERα) and form the HDAC3-ERα complex, which suppressed selective apoptosis mediated by TNF-α in MCF-7 cells, and the process is dependent on caspase-7 (98).

Other publications also reported the function of p53 in HDACi-mediated apoptosis. The HDACi-induced process includes activating p53, whereas p53 was not found to be a prerequisite for anticancer processes (175). A majority of studies have shown that HDACis function without p53 since their anticancer effects do not fluctuate with cell p53 status (176). Despite this, other studies have concluded that p53 plays a crucial role in inducing tumour cells in response to HDACi treatment. To test this hypothesis, scientists have generated isogenic HCT-116 colon tumour cells with different p53 statuses. Vorinostat, apicidin, and VPA can function efficiently regardless of the level of p53. Nevertheless, entinostat is influenced by p53 status to a certain degree (175). This molecule can inhibit [FADD]-like interleukin-1 β-converting enzyme inhibitory protein [FLIP(L)] and thus promote p53-induced apoptosis (177).

Moreover, HDAC1 and HDAC8 simultaneously repress BMF. The HDAC8 inhibitor methylselenopyruvate was found to promote the transcription of BMF and apoptosis induced by BMF in colon cancer cells (178). Recently, HDAC11 was shown to be important for the proliferation of oncogenic JAK2-driven myeloproliferative neoplasms, and downregulation of HDAC11 expression could promote apoptosis in human leukaemia (70).

### DNA Damage Response

A growing body of research has shown that HDACs are involved in DNA damage repair (DDR) responses. HDACs serve as protective agents against DNA damage due to their necessary role in remodelling chromatin and balancing the acetylation of proteins associated with DNA damage (179).

HDAC1, along with HDAC2, binds to DNA damage regions to deacetylate histone proteins at H3K56 and H4K16 and accelerates nonhomologous end joining (NHEJ) pathways, thereby promoting double-strand break (DSB) repair (180). For HDAC3, inactivating HDAC3 leads to genomic instability, and HDAC3 deficiency in the liver contributes to HCC (181). More specifically, HDAC3 deacetylase acts on and stabilizes Tip60, thereby reducing apoptosis triggered by DNA damage (182). Furthermore, HDAC inhibition blocks DSB repair and sensitizes tumour cells to ionizing radiation and DNA damage agents.

HDAC3 was also reported to participate in nucleotide excision repair (NER). HDAC3 acts on H3K14 after ultraviolet irradiation and promotes XPC recruitment to DNA-damaged sites, thereby exerting a positive effect on the global genomic NER (183).

Several class II HDACs are involved in DNA damage repair. For example, HDAC4 and 53BP1 colocalize in nuclear foci after DSBs. Knocking down HDAC4 resulted in low levels of 53BP1 and inactivation of the DNA damage-induced G2 checkpoint (184). HDAC9 and HDAC10 were shown to play roles in homologous recombination (153, 185). Furthermore, Doctor Zhang reported that the DNA mismatch repair protein MutS protein homologue 2 (MSH2) is associated with class IIb HDACs. Specifically, HDAC6 functions as an MSH2 inhibitor *via* deacetylation and ubiquitination (186).

Sirtuins interact with many DDR proteins, such as Ku70, NBS1, hMOF, WRN, APE1, XPA, PARP-1, TopBP1, and KAP1, thus regulating several DDR pathways (187). In tumour cells, SIRT1 was reported to prevent p53 acetylation and thus promote cell survival after DNA damage (156, 188). Therefore, knocking down SIRT1 exerts positive feedback in cancer treatment, revealing that SIRT1 is an potential target for cancer treatment. However, when cancer cells suffer severe damage, SIRT1 is repressed due to phosphorylation at Ser682 by the DNA damage-responsive kinase HIPK2 (189). This process results in cell death under severe DNA damage. SIRT1 plays a crucial role in supporting genome integrity and stability (190). Thus, enhancing SIRT1 in this manner may be a potential therapeutic strategy.

SIRT6 also plays a notable role in DNA repair. This molecule was first reported to abrogate genome instability through the alteration of DNA base excision repair. SIRT6 can be directly recruited to DNA damage sites and enhance mono-ADP-ribosylation of PARP1, thereby facilitating DSB repair (143), which is achieved through phosphorylation of SIRT6 by JNK (191). To suppress genomic instability, SIRT6 recruits and deacetylates the ISWI-chromatin remodeler SNF2H at histone H3K56 (192). A recent study showed that inhibition of HDAC8 or SIRT6 induces DNA repair deficiencies in homologous recombination and NHEJ pathways in leukaemia-initiating cells, and such DNA repair deficiencies are synergistic with nicotinamide phosphoribosyl transferase (NAMPT) targeting (193). Overall, we found that some class III HDAC members, due to their deacetylase activity, are essential in the DNA damage response.

### Metastasis

EMT is an important step in tumour cell invasion and metastasis. Many studies have shown that HDACs can regulate EMT in diverse cancer types. The most important feature of EMT is downregulated E-cadherin (encoded by CDH1) expression. Some CDH1 transcriptional inhibitors, such as Snail, Slug, Twist, and ZEB1/2, have been identified (194–196).

HDACs bind to the CDH1 promoter and deacetylate H3 and H4 histones. In pancreatic cancer, the Snail/HDAC1/2 complex was shown to repress CDH1, causing a reduction in E-cadherin expression and subsequently inducing EMT (197). The complex mentioned above was also found to can interact with EZH2 and cause CDH1 silencing (198). Additional studies reported that ZEB1 induces the binding of HDACs with the CHD1 promoter in human pancreatic cancers (199). Furthermore, ZEB1 and HDACs can modify the splicing of CDH1 exon 11. Therefore, the reduction in CDH1 expression is a comprehensive outcome caused by transcriptional inhibition and abnormal splicing (200). Mocetinostat, an HDACi, efficiently reduced the expression of ZEB1 in pancreatic tumour cells (87), revealing a therapeutic effect of class I HDACis in treating EMT and metastasis of cancers.

A recent study demonstrated that class I HDACs are related to maspin repression, which is often detected in prostate cancer. Inhibiting class I HDACs facilitated maspin re-expression, thus suppressing the proliferation and migration of prostate tumour cells (201). In addition, in colorectal cancers, HDAC3 was recruited to Runx 2 and repressed metastasis (202).

HDAC7 was reported to enhance EphA2 expression by downregulating miR-4465 expressing, showing a positive effect on tumour proliferation, migration, and invasion in nasopharyngeal carcinoma (NPC) (203). HDAC11 can upregulate the expression of RRM2, a gene associated with promigratory and metastatic phenotypes in diverse cancers, thus promoting metastasis of tumour cells (109).

The function of SIRT1 in the EMT process is associated with tumour types. In prostate cancer cells, SIRT1 and ZEB1 simultaneously bind to CDH1, thereby silencing transcription, which results in metastasis (204). In addition, the MPP8-SIRT1 interaction is considered to play a crucial role in CDH1 repression in prostate cancer cells (205). In melanoma, SIRT1 can deacetylate Beclin-1, thereby leading to E-cadherin in autophagy, and a low level of E-cadherin results in an enhanced EMT process (206). In colorectal cancer, EMT is activated by SIRT1 by upregulating Fra-1 expression (207). However, SIRT1 was suggested to reduce metastasis through deacetylation of Smad4 and repression of TGF-β-triggered signalling. The latter could affect matrix metalloproteinase-7 (MMP-7) in breast and oral cancer cells (208, 209). SIRT2 was reported to deacetylate Slug and stabilize its protein to enhance EMT (210). Thus, Sirtuins play important roles in metastasis.

### Angiogenesis

Angiogenesis is critical in tumour growth and metastasis. Hypoxia or a hypoxic microenvironment enables the initial stage of angiogenesis, and its process is mainly controlled by hypoxia-inducible factor 1α (HIF-1α). HDACs such as HDAC1 function explicitly as deacetylase enzymes of HIF-1α, thereby preventing HIF-1α degradation. Abnormal expression of HDAC1 leads to overexpression of HIF-1α and VEGF in tumours, which subsequently promotes angiogenesis (211). Consistent with this conclusion, HDACis can lead to HIF-1 and VEGF degradation and repression (211). HDAC4, HDAC6, HDAC10, and SIRTs show similar mechanisms, as they promote angiogenesis by enhancing VEGF, EGF, and HIF 1α levels (157).

HDAC4, HDAC5, and HDAC6 act as mediators of HIF-1 activity by promoting the recruitment of obligatory cofactors, such as HSP90 and p300, thereby enhancing its transcriptional activity (212). Moreover, SIRT1 was found to play an opposite role, since it deacetylases HIF-1α and prevents HIF-1α from interacting with p300, resulting in a reduction in HIF-1α activity. SIRT1 inhibition in the hypoxic microenvironment maintains high HIF-1 activity (213).

However, HDAC5 and HDAC6 also have antiangiogenic roles since they can deacetylate HIF-1a chaperones, namely, HSP70 and HSP90. Suppressing HDAC5 and HDAC6 causes substantial acetylation of these proteins, and thus, HIF-1 is in an immature form and easily degrades (214). In endothelial cells, HDAC5 inhibits the expression of proangiogenic genes, including FGF2 or Slit2 (215). HDAC5 can suppress cysteine-rich angiogenic inducer 61 (CYR-61), which is an antifibrotic and proangiogenic mediator, to repress angiogenesis (216). HDAC6 also has a positive effect on angiogenesis through deacetylation of the actin-remodelling protein cortactin and thus induces migration and sprouting in endothelial cells (217).

Overall, HDACs function by regulating various pro- and antiangiogenic proteins in angiogenesis, revealing that they may be promising targets for cancer therapy. HDAC inhibition is considered to exert antiangiogenic effects by downregulating the expression of proangiogenic genes.

### Autophagy

Autophagy exerts a dual role in tumorigenesis. Autophagy can eliminate damaged subcellular fractions, thereby preventing the transformation of normal cells into tumour cells (218). Therefore, the deletion of autophagic proteins was found to facilitate tumorigenesis. However, autophagy also promotes survival of cancer cells under metabolic stress, which may result in resistance to anticancer treatment (219).

Many HDAC enzymes show dual effects in the autophagic process. Class I type HDACs are thought to be conducive to autophagic flux in mice (220). Knocking down HDAC1 and HDAC2 was reported to impede autophagic flux (221). HDAC2 can directly function on the SNARE domain of syntaxin 17 (STX17) during autophagy. Deacetylated STX17 interacts with SNAP29 and HOPS, thus promoting the fusion of autophagosomes with lysosomes (222).

HDAC4 and HDAC5 are considered to alter autophagic flux while functioning as positive regulators of tumour cell growth. HDAC6 is needed to clear misfolded proteins by inducing autophagy (66) and enhances autophagy through its relationship with microtubule proteins (223). HDAC6 possesses a ubiquitin-binding domain, which is involved in responding to cytotoxic protein aggregates (224). In neurodegenerative diseases and cerebral ischaemia, HDAC6 plays an intermediary role between autophagy and the ubiquitin proteasome system (UPS). Specifically, autophagy can be strongly promoted and serve a compensatory role for HDAC6 under UPS damage (225, 226). HDAC6 plays an essential role in ubiquitin-selective quality control (QC) autophagy, rather than starvation-induced autophagy (227), in HDAC6 knockout mouse embryonic fibroblasts (MEFs). QC autophagy is reasonably distinct from starvation-induced autophagy due to the participation of ubiquitinated substrates and ubiquitin-binding HDAC6 and p62 (228). Furthermore, mitochondria have a selective method of elimination similar to autophagy. Specifically, parkin-mediated mitochondrial ubiquitination can also recruit the autophagic components HDAC6 and p62, which facilitate autophagic initiation and progression in impaired mitochondria (228). Despite p62’s passive role in autophagy as a substrate receptor, a recent study indicated that it can also act positively as an autophagic suppressor by promoting HDAC6 expression and subsequently recruiting deacetylated α-tubulin and unstable microtubules, resulting in dysfunctional autophagic flux, in prostate cancer cells (229). In neuroblastoma, loss of HDAC10 caused autophagosome/lysosome fusion blockade and autophagic flux arrest, contributing to enhanced cell sensitivity to chemotherapy (106). HDAC10 can also deacetylate HSP70 protein families, which may be linked to autophagy-mediated cell survival (106). Thus, class IIb HDACs are likely to primarily modulate autophagic flux by influencing autophagosome-autolysosome fusion.

Sirtuins are also involved in the regulation of the autophagic process. SIRT1 showed different effects in different cell lines and has a dual function in autophagy (230). SIRT1 is required to trigger starvation-induced autophagy since it affects Atg5, Atg7, Atg8, and LC3, essential members of the autophagic process (231, 232). Moreover, forkhead box O3 (FOXO3) can be deacetylated by SIRT1 and subsequently upregulates the expression of numerous autophagic genes. Additional studies have revealed that FOXK1/2, counterbalancing factors of FOXO3, recruit SIN3A-HDAC complexes to interfere with the acetylation of histone H4 and the expression of crucial autophagic genes (233). In addition, SIRT1 was shown to influence the PI3K/Beclin 1 and mTOR pathways in embryonic stem cells (ESCs), which in turn affects oxidative stress-induced autophagy (234). Unlike SIRT1, SIRT3 and SIRT5 were reported to participate in not only late but also early autophagic regulation (230). SIRT3 can act on mitochondria and trigger clearance under oxidative stress or starvation conditions (235). SIRT3 can also regulate the phosphorylation of activated protein kinase (AMPK) to enhance autophagy (236, 237). SIRT5 deacetylates lactate dehydrogenase B (LDHB) and increases its enzymatic activity. Protons (H) generated by LDHB contribute to autophagy in cancer cells (238). SIRT5 was also reported to participate in ammonia-induced autophagy, which is achieved through alteration of glutamine metabolism (136). SIRT2 dissociates from FOXO1 under stress conditions and drives the latter into a hyperacetylated state, which facilitates the autophagic process (136). SIRT6 was also reported to induce autophagy by restricting the transcription of the transcriptional repressor Nkx3.2, thus enhancing GATA5 expression (239).

In summary, the HDAC context-dependent functions in autophagic processes contribute to cancer treatment by targeted therapy.

### HDAC and Immunity

HDAC3 is involved in lipopolysaccharide (LPS)-directed cytokine secretion in monocytes and M1 macrophages. HDAC3 disrupts the process by which inflammation-activated M1 macrophages mediate LPS tolerance (240).

Inhibition of HDAC mediates tumour-associated macrophages to specify the antitumour phenotype, resulting in decreased immune suppression and increased antitumour immune responses (241). Retinoic acid-related orphan receptor α (RORα)/HDAC-directed inhibition of NF-κB signalling modulates cholesterol metabolism in CD8+ T cells, which may provide a new therapeutic target in cancers such as colon tumours (242). Research has shown that suppression of HDACs is involved in restoration of IFN signalling, leading to enhancement of long-term antitumour immunity and repression of prostate tumour growth in bone (243). Moreover, Tcf1-instrinsic HDAC activity participates in suppressing excessive CTLA4 induction in protein immunization-elicited T cells, therefore protecting B-cell functions (244).

### HDAC and the Tumour Microenvironment

Suppression of HDACs is involved in regulating infiltrating macrophages and repressing the trafficking of myeloid-derived suppressor cells into tumours, thereby enhancing T cell activation in the tumour microenvironment (241). Suppression of HDACs plays an antitumour role in many cancers, such as pancreatic tumours, colorectal tumours and NSCLC, which can mediate tumour microenvironment changes, therefore enhancing the antitumour function of anti-PD-1 antibodies (245). Inhibition of HDACs can promote the expression of antigen-presenting machinery genes and CTL infiltration. Research has shown that HDACs are involved in regulating the tumour microenvironment, therefore regulating the immunotherapy response (246). Anne and her colleagues found that HDAC is involved in the transition of the tumour microenvironment from “cold” to “hot”, thereby inhibiting immune checkpoint blockade therapies (247).

## Conclusion

Over the years, HDACs have been intensively investigated. To date, HDACs have been declared a key driver of cancers by modulating the dynamic acetylation of histone and nonhistone cellular substrates. As important regulators of histone acetylation clearance, HDACs exhibit abnormal expression and functions in cancer, indicating they are promising targets. Nevertheless, the precise mechanism of HDACs as tumorigenic agents is still worth studying. In many types of tumours knocking out HDACs was shown to lead to cell cycle arrest or apoptosis. In addition, the antitumour effect of HDACs was detected in a specific cellular setting. Selective HDACis serve as effective tools in elucidating the roles of HDACs. Moreover, additional studies are needed to systematically determine how each HDAC functions in specific conditions.

## Author Contributions 

RH wrote the manuscript. LH completed the English revision. GY provided constructive feedback and guidance. GS completed critical revisions and proofread the manuscript. All authors contributed to the article and approved the submitted version.

## Funding

This work was supported by the National Natural Science Foundation of China under Grant Number 81572900, The Fundamental Research Funds for the Central Universities of Central South University under Grant Number 2020105330104, and the National Key R&D Program of China, Stem Cell, and Translation Research under Grant Number 2016YFA0102000.

## Conflict of Interest

The authors declare that the research was conducted in the absence of any commercial or financial relationships that could be construed as a potential conflict of interest.

## Publisher’s Note

All claims expressed in this article are solely those of the authors and do not necessarily represent those of their affiliated organizations, or those of the publisher, the editors and the reviewers. Any product that may be evaluated in this article, or claim that may be made by its manufacturer, is not guaranteed or endorsed by the publisher.

## Abbreviations

AMPK, activated protein kinase; APL, acute promyelocytic leukaemia; Bcl-2, B-cell lymphoma-2; CDK, cyclin-dependent kinase; CLL, chronic lymphocytic leukaemia; CREB, cAMP-response element-binding protein; CTCL, cutaneous T cell lymphoma; DSB, DNA double-strand break; E2F, Early 2 factor; ELM2, egl-27 and MTA1 homology domain 2; ERα, oestrogen receptor-α; FOXO, forkhead box O; GLS, mitochondrial glutaminase; HATs, histone acetyltransferases; HCC, hepatocellular carcinoma; HDAC, histone deacetylases; HIF-1, hypoxia-inducible factors 1; HL, Hodgkin’s lymphoma; ISWI, imitation switch; LDH, lactate dehydrogenase; LSD1, lysine-specific demethylase 1 proteins; MC-LR, microcystin-leucine arginine; MMPs, matrix metalloproteinases; MPNs, myeloproliferative neoplasms; MPP8, M-phase phosphoprotein 8; MSH2,MutS protein homologue 2; NER, nucleotide excision repair; NHEJ, nonhomologous end joining pathway; NICD1, Notch1 intracellular domain; NuRD, nucleosome remodelling and deacetylase; OSCC, oral squamous cell carcinoma; PHF23, plant homeodomain finger protein 23; PML-RAR, promyelocytic leukaemia-retinoic acid receptor; Rb, retinoblastoma protein; SANT, Swi3, Ada2, NCoR and TFIIIB domain; SDS3, suppressor of defectivesilencing3; SIN3A, switch-independent protein 3A; STX17, syntaxin 17; T-ALL, T cell acute lymphoblastic leukaemia; UPS, ubiquitin proteasome system; XPC, xeroderma pigmentosum complementation group C.
